# Supplementary material for: The interactome of intact mitochondria by cross-linking mass spectrometry provides evidence for coexisting respiratory supercomplexes
Source: Mol Cell Proteomics. 2017 Dec 8;17(2):216–32. doi: 10.1074/mcp.RA117.000470 (PMC5795388; doi:10.1074/mcp.RA117.000470)
Supplement: Supplemental Data [file supp_RA117.000470_133922_0_supp_23978_fzffwf.zip › spectra_annotation/mito_spectra_annotation/12-1-2-1-12-1.pdf]

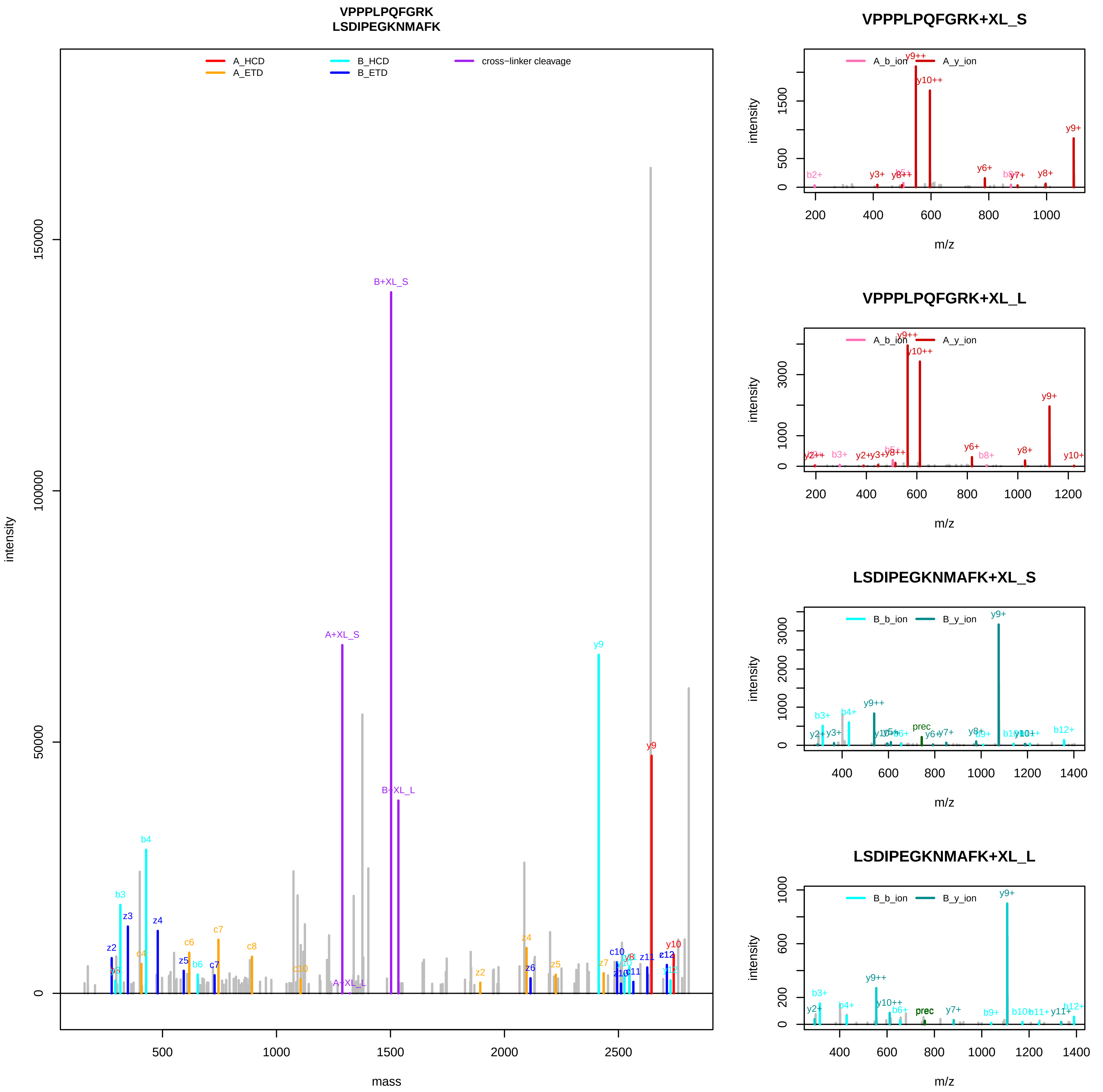

VPPPLPQFGRK
LSDIPEGKNMAFK
A_HCD
A_ETD
B_HCD
B_ETD
cross−linker cleavage
B+XL_S
A+XL_S
B+XL_L
A+XL_L
y9
y10
b4
b3
z2
z3
z4
z5
c4
c6
c7
c8
c10
b6
z6
z7
c10
c11
c12
z10
z11
y12
intensity
mass
0
50000
100000
150000
500
1000
1500
2000
2500
VPPPLPQFGRK+XL_S
A_b_ion
A_y_ion
y9++
y10++
y9+
y6+
y8+
y3+
b2+
m/z
VPPPLPQFGRK+XL_L
y10+
LSDIPEGKNMAFK+XL_S
B_b_ion
B_y_ion
prec
y9++
y9+
b3+
b4+
b12+
LSDIPEGKNMAFK+XL_L
